# Chili Pepper Object Detection Method Based on Improved YOLOv8n

**DOI:** 10.3390/plants13172402

**Published:** 2024-08-28

**Authors:** Na Ma, Yulong Wu, Yifan Bo, Hongwen Yan

**Affiliations:** College of Information Science and Engineering, Shanxi Agricultural University, Jinzhong 030801, China; manasxau@163.com (N.M.); 17652525251@163.com (Y.W.); 13453109054@163.com (Y.B.)

**Keywords:** chili pepper, YOLOv8, object detection, lightweight, ablation experiment

## Abstract

In response to the low accuracy and slow detection speed of chili recognition in natural environments, this study proposes a chili pepper object detection method based on the improved YOLOv8n. Evaluations were conducted among YOLOv5n, YOLOv6n, YOLOv7-tiny, YOLOv8n, YOLOv9, and YOLOv10 to select the optimal model. YOLOv8n was chosen as the baseline and improved as follows: (1) Replacing the YOLOv8 backbone with the improved HGNetV2 model to reduce floating-point operations and computational load during convolution. (2) Integrating the SEAM (spatially enhanced attention module) into the YOLOv8 detection head to enhance feature extraction capability under chili fruit occlusion. (3) Optimizing feature fusion using the dilated reparam block module in certain C2f (CSP bottleneck with two convolutions). (4) Substituting the traditional upsample operator with the CARAFE(content-aware reassembly of features) upsampling operator to further enhance network feature fusion capability and improve detection performance. On a custom-built chili dataset, the F_0.5-score_, mAP_0.5_, and mAP_0.5:0.95_ metrics improved by 1.98, 2, and 5.2 percentage points, respectively, over the original model, achieving 96.47%, 96.3%, and 79.4%. The improved model reduced parameter count and GFLOPs by 29.5% and 28.4% respectively, with a final model size of 4.6 MB. Thus, this method effectively enhances chili target detection, providing a technical foundation for intelligent chili harvesting processes.

## 1. Introduction

Chili peppers are important agricultural products and economic crops [1,2,3]. China is a major producer of chili peppers, with the planting area accounting for 8–10% of the national vegetable planting area annually, with an output value of approximately 250 billion yuan, providing significant economic and social benefits [4,5,6]. Currently, all links in the chili pepper industry, except for harvesting, have been mechanized. Faced with social issues such as an aging population and labor shortages, the development of intelligent harvesting equipment is particularly important. With the advancement of information technology, traditional agriculture is moving towards intelligent development, providing a direction for the upgrading of the chili pepper industry. Target detection of chili pepper fruits can provide necessary technical support and intelligent solutions for the chili pepper harvesting process.

Early detection of peppers mainly used traditional machine vision. Li et al. [7] extracted color and shape features of dried peppers using the line sectioning method and minimum bounding rectangle algorithm, proposed segmentation of multiple dried pepper images based on the watershed algorithm, and used the naive Bayes algorithm for grading multiple dried peppers, achieving a classification accuracy of over 95%. Hendrawan et al. [8] used color and texture feature methods to identify the total content of carotene in peppers, with a mean square error (MSE) accuracy of 80%. Azis et al. [9] adopted digital image processing technology to accurately classify the quality of dried peppers based on physical features such as length and color of the peppers, achieving a classification accuracy of 94.4%. Sajjan et al. [10] used RGB, HSI, and L*a*b* color model features to identify four different varieties of peppers with and without stems. The recognition rate of peppers with stems based on RGB color features was 70%, while for peppers without stems was 85%. The recognition rate based on HSI color features for peppers with stems was 80%, and for peppers without stems was 90%. The recognition rate based on L*a*b* color features for peppers with stems was 85%, and for peppers without stems was 95%. From the above research, it can be seen that traditional digital image processing techniques often require manual feature extraction in object recognition, which is time-consuming and labor-intensive. Additionally, these techniques are susceptible to background, lighting, and human factors, leading to lower recognition accuracy.

In recent years, pepper object detection has mainly focused on pepper grading and sorting processes. The identification of pepper fruits under natural conditions is challenging due to complex environments, diverse lighting conditions, and significant occlusion. With the development of the Internet of Things, big data, AI, and other technologies, non-contact object detection based on machine vision and deep learning has laid the foundation for intelligent harvesting of agricultural crops [11,12,13,14]. Zhang et al. [15] proposed using the RS loss function to replace the cross-entropy function in RPN and the Soft-NMS algorithm to replace the original NMS algorithm to improve Faster R-CNN. Experimental results showed that the improved Faster R-CNN achieved the mAP of 90.56% for identifying yellow leaf diseases. Liu et al. [16] introduced an improved method for detecting sprouting and surface damage of potatoes based on Faster R-CNN. By replacing the feature extraction network in Faster R-CNN with the ResNet50 residual network and designing a feature pyramid network (FPN) integrated with ResNet50 to increase network depth, the improved model achieved an mAP of 98.89% for potato detection, 97.52% for sprouting detection, and 92.94% for surface damage detection. Li et al. [17] proposed an enhanced Faster R-CNN fruit detection and localization method by introducing an efficient channel attention (ECA) mechanism and a multi-scale fusion feature pyramid (FPN). Experimental results showed that the improved model had average precision values of 96.16% and 86.95% for graspable and non-directly graspable apples, respectively, with an overall average precision of 92.79%, representing a 15.68 percentage point increase over traditional Faster R-CNN. Xie et al. [18] presented a method for single-tree crown extraction from drone images based on Mask RCNN and transfer learning, achieving an overall accuracy of 93.59%. Guan [19] proposed an improved Mask R-CNN model to enhance the accuracy and reliability of cow face detection. Compared with the original model, the improved Mask R-CNN model with MResNet, ResNet101, and ResNet50 reduced the missed detection rates by 7.70%, 6.32%, and 4.81%, and reduced the false alarm rates by 3.68%, 3.61%, and 1.71%, respectively.

The above all used two-stage algorithms similar to RCNN, which have high detection accuracy, but due to the detection process being divided into two stages, the speed is relatively slow. Another type of one-stage network represented by SSD, YOLO, etc., has a faster detection speed due to the characteristics of one-stage detection. Zhang et al. [20] used an improved YOLOv5 algorithm to recognize cherry fruits in natural environments. Compared to other existing models and the YOLOv5 model improved with a single structure, the proposed comprehensive improvement model has higher detection accuracy, increasing the mean average precision by 2.9 percentage points. Liu et al. [21] borrowed from human pose detection algorithms to establish the improved YOLOv8-Pose model for identifying and detecting stem key points of ripe strawberries. The mAPkps of improved YOLOv8-Pose under occlusion, lighting, and angle conditions are 94.52%, 95.48%, and 94.63%, respectively. Li et al. [22] proposed a chestnut recognition method based on the improved YOLOv8 model called YOLOv8-PBi. Experimental results showed that the improved YOLOv8-PBi model has an accuracy, recall, and mAP of 89.4%, 74.9%, and 84.2%, respectively; compared to the original base network YOLOv8s, the model weight decreased by 46.22%. Liu et al. [23] built a pepper recognition and positioning system based on the YOLOv3 model and a realsense depth camera, with a recall of 98%, mAP of 95%, and precision of 85.4%.

Currently, there is still relatively little research on the automated detection of peppers. However, the YOLO algorithm has successfully been applied in agricultural target detection due to its excellent detection accuracy and speed [24,25,26,27]. To better deploy detection algorithms on edge devices with limited computing power, network structures can be modified to further reduce model parameters and improve detection speed. The YOLOv8 algorithm [28,29,30], characterized by small average weight files, short training times, and fast inference speeds, demonstrates high efficiency and accuracy in object recognition and localization. Therefore, this paper bases its model on YOLOv8n, first introducing the improved HGNetV2 [31] model to replace the YOLOv8 backbone network. Next, the SEAM (spatially enhanced attention module) [32] is integrated into the YOLOv8 detection head, followed by optimization of the C2f network structure using the dilated reparam block module [33]. Finally, the CARAFE [34] upsampling operator replaces the traditional upsampling operator in YOLOv8 to enhance feature extraction capabilities. These lightweight improvements aim to reduce computational requirements, conserve device resources, and meet the pepper detection demands during mechanized harvesting under natural conditions.

## 2. Results and Analysis

### 2.1. Analysis of Comparative Results of YOLO-Series Algorithms

To validate the performance of the baseline network YOLOv8n in pepper object detection, metrics such as precision, recall, F_0.5-score_, mAP_0.5,_ mAP_0.5:0.95_, model size, GFLOPs, and parameter count were used as fundamental evaluation criteria. YOLOv8n was compared with YOLO series models YOLOv5n, YOLOv6n, YOLOv7-yiny, YOLOv9, and YOLOv10, as well as Faster R-CNN and RT-DETR algorithms under the same conditions. The experimental results are shown in Table 1. According to the results in Table 1, YOLOv5n exhibits the smallest model size, GFLOP count, and parameter count, but its F_0.5-score_, mAP_0.5_, and mAP_0.5:0.95_ are relatively low. YOLOv6n exhibits higher precision. Although RT-DETR has the highest mAP_0.5_ value, it also has a larger model size, GFLOP count, and parameter count. YOLOv8n outperforms the other seven object detection networks in F_0.5-score_, and also has a smaller model size, GFLOP count, and parameter count. Therefore, from an overall performance perspective, YOLOv8n was selected as the baseline network for this study, aiming to further lighten the model and improve detection accuracy.

### 2.2. Performance of Improved YOLOv8

**Figure 1:**
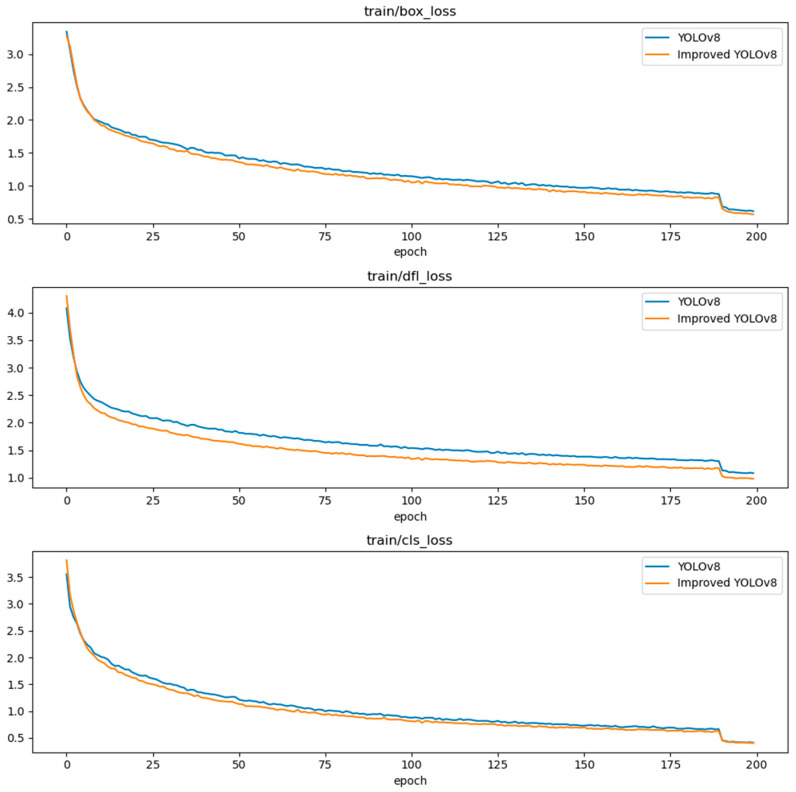
Comparison of loss curves for improved YOLOv8 and YOLOv8n.

We compared the loss curves during the training of the improved YOLOv8 and the baseline YOLOv8 algorithm for pepper object detection, as shown in Figure 1. It can be seen from the figure that the loss curve of the improved YOLOv8 in this paper is lower than that of the baseline YOLOv8n, gradually decreasing and stabilizing over 200 runs. This indicates that the improved YOLOv8 in this paper can accelerate the model’s convergence faster and extract features more effectively.

The specific experimental results of the improved YOLOv8 and YOLOv8n are shown in Table 2. Compared to the baseline YOLOv8n algorithm, the improved algorithm in this paper shows an increase of 2.5, 0.1, 1.98, 2, and 5.2 percentage points in P, R, F_0.5-score_, mAP_0.5_, and mAP_0.5:0.95_, respectively. The model file size is 4.6 MB, approximately 70% of the size of the baseline YOLOv8 model. Additionally, the improved model reduces the number of parameters and GFLOPs by 29.5% and 28.4%, respectively. These improvements significantly enhance detection accuracy while reducing computational and parameter overhead, demonstrating the effectiveness of the algorithm proposed in this paper.

**Figure 2:**
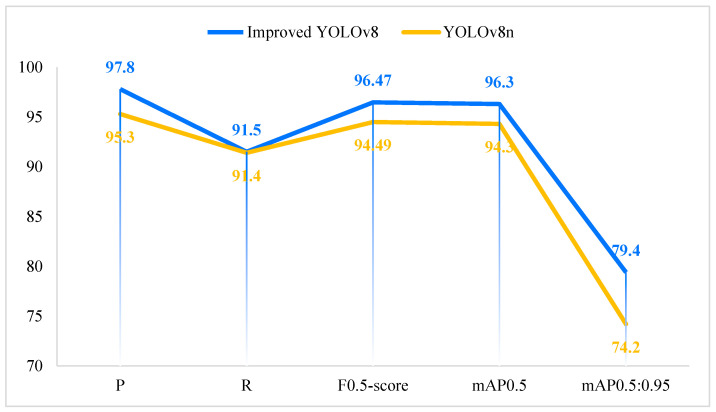
Detection accuracy comparison for improved YOLOv8 and YOLOv8.

**Figure 3:**
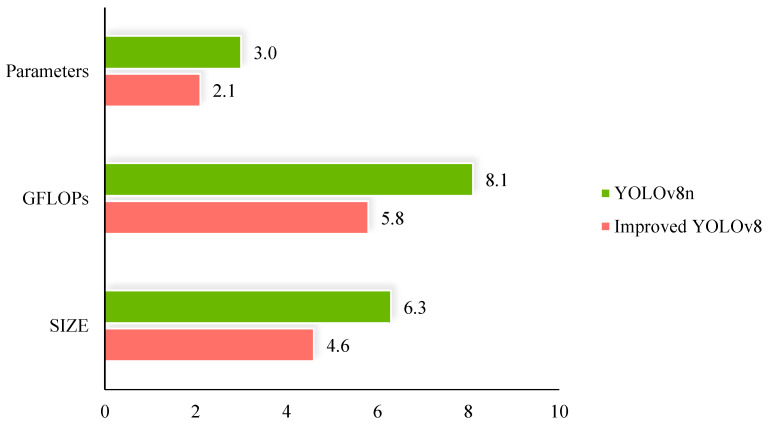
Comparison of weight for improved YOLOv8 and YOLOv8.

We plotted comparative graphs between the improved algorithm proposed in this paper and the baseline YOLOv8n algorithm in terms of detection accuracy and model lightweighting, as shown in Figure 2 and Figure 3. Figure 2 illustrates that the improved algorithm in this paper outperforms the baseline YOLOv8n algorithm in terms of P, R, F_0.5-score_, mAP_0.5_, and mAP_0.5:0.95_. Figure 3 demonstrates that the improved algorithm in this paper excels over the baseline YOLOv8n algorithm in terms of parameter count, GFLOPs, and model size.

### 2.3. Ablation Experiments

To validate the effectiveness of each improvement module for chili detection, experiments were conducted by ablating each module based on YOLOv8n as the baseline network. The evaluation primarily focused on accuracy metrics, including F_0.5-score_, mAP_0.5_, and mAP_0.5:0.95_.

The results of the ablation experiments are shown in Table 3. “√ ” means that the model contains this module, while “×” means it does not. In Table 3, Experiment 1 represents the baseline network YOLOv8’s performance in chili pepper detection. Experiments 2–5 show the chili pepper detection results after applying various improvements to the baseline YOLOv8 network. From Experiment 2, it is observed that replacing the baseline YOLOv8 backbone network with the improved HGNetV2 network enhances feature fusion capability and detection speed. However, the F_0.5-score_ decreased while the mAP_0.5_ and mAP_0.5:0.95_ increased by 1.9 and 3.7 percentage points, respectively. The parameter count, model size, and GFLOPs were all reduced. This is due to the improved HGNetV2 network potentially losing some chili pepper features while effectively reducing model weights and computational redundancy, leading to a slight decrease in the F_0.5-score_. Experiment 3 shows that introducing the SEAM attention mechanism into the baseline YOLOv8 detection head improves both mAP and mAP_0.5:0.95_, while reducing the model’s parameter count, size, and GFLOPs. Experiment 4 demonstrates that optimizing parts of C2f in the baseline YOLOv8 improves both detection accuracy and speed. The optimized C2f structure reduces model parameters, speeds up detection, and better focuses on target features. Experiment 5 indicates that replacing the traditional upsampling method in the baseline YOLOv8 with CARAFE upsampling increases computational load but improves network feature fusion capability, with enhancements in F_0.5-score_, mAP_0.5_, and mAP_0.5:0.95_. In summary, applying the improved HGNetV2 network, SEAM attention mechanism, optimized C2f, and CARAFE upsampling methods individually to the baseline network results in improvements in detection accuracy or model lightweighting.

To further enhance network performance, Experiments 6, 7, and 8 applied the four improvement methods to the baseline YOLOv8 using different approaches. Experiment 6 incorporated both the improved HGNetV2 network and the SEAM attention mechanism. Comparing the results of Experiment 6 with Experiment 2 shows that adding the SEAM attention mechanism further improves feature fusion capability and detection speed, with the F_0.5-score_ rising by 1.82 points, mAP_0.5_ increasing by 0.5 points, and mAP_0.5:0.95_ rising by 1.7 points. The parameter count decreased by 8%, model size dropped from 5.0 M to 4.7 M, and GFLOPs increased by 15.9%. This indicates a mutually beneficial effect of the improved HGNetV2 network and the SEAM attention mechanism, leading to enhanced detection accuracy and model lightweighting. Experiment 7 involved using the improved HGNetV2 network, SEAM attention mechanism, and optimized C2f. The results show that using DRB to improve the C2f structures at layers 16, 19, and 22 enhances detection speed, reduces model parameters by 8.4%, decreases model size from 4.7 M to 4.3 M, and lowers GFLOPs from 4.7 to 4.3. Although mAP_0.5_ and mAP_0.5:0.95_ slightly decreased, the F_0.5-score_ increased by 0.88 points. This indicates that optimizing the C2f structure significantly impacts model lightweighting but has a limited effect on enhancing feature fusion capability in the current network. Experiment 8 applied all four improvements—improved HGNetV2 network, SEAM attention mechanism, optimized C2f, and CARAFE upsampling—to the baseline YOLOv8. The results reveal that replacing traditional upsampling with CARAFE upsampling, while increasing computational load, improves feature fusion capability, with F_0.5-score_ rising by 0.13 points and mAP_0.5:0.95_ increasing by 1.1 points.

In summary, the improved YOLOv8n object detection network achieves improvements in F_0.5-score_, mAP_0.5_, and mAP_0.5:0.95_ on chili data, with increases of 1.98, 2, and 5.2 percentage points, respectively. This is accompanied by reductions in computational load, model weight, and parameter count, achieving model lightweighting.

### 2.4. Comparison of Other Backbone Network Replacements

This experiment is based on the YOLOv8 object detection network, replacing mainstream feature extraction backbone networks such as EfficientViT, Fasternet, and EfficientFormerV2. By keeping all parameters except the backbone network consistent, the effect of different backbone networks on target training is compared.

According to Table 4, the use of the HGNetV2 improvement method—compared to EfficientViT, Fasternet, and EfficientFormerV2, although this method is not optimal in terms of training precision, recall, and mAP—has the best lightweight effect. The model’s weight, number of parameters, and GFLOPs are the smallest, making it better suited for deployment requirements.

### 2.5. C2f Optimization Results and Analysis

To verify the improvements to C2f in this experiment, C2f optimization trials were conducted. We enhanced C2f using DilatedReparamBlock (DRB), DiverseBranchBlock (DDB), ODConv, and FasterBlock from FasterNet as shown in Table 5. Trial 1 represents the model without C2f optimization. Trials 2, 3, 4, and 5 represent different methods for optimizing the C2f module across 16 layers. The results showed that these optimizations led to varying reductions in model parameters and GFLOPs compared to the original model. However, their impact on F_0.5-score_ differed: notably, the DRB module showed the most significant improvement, increasing F_0.5-score_ by 0.54 percentage points. Trials 6 and 7 further validated the effect of optimizing different positions of the C2f module using the DRB module. Trial 6 optimized C2f modules at layers 16 and 19 with DRB, while Trial 7 optimized C2f modules at layers 16, 19, and 22 with DRB. Results indicated that increasing the number of optimized C2f modules led to continued reductions in model parameters and GFLOPs. Optimizing C2f modules at layers 16, 19, and 22 achieved the best F_0.5-score_ of 96.34%, an increase of 0.88 percentage points.

### 2.6. Model Visualization

**Figure 4:**
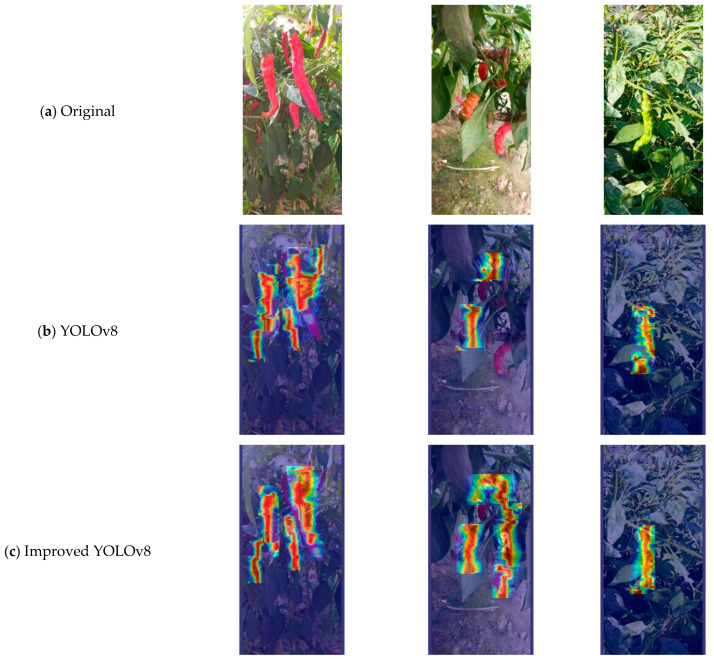
Model heat map visualization.

To more intuitively observe the improvement of the model in chili pepper target recognition ability in this paper, Grad-CAM (gradient-weighted class activation mapping) [35] was used to generate heat maps. Grad-CAM utilizes backpropagation of the training weights, performing spatial dimension global average pooling on the obtained gradient matrix, and weighting and activating each channel of the feature layer to generate the heat map. The brightness of a specific area in the heat map can indicate the parts of the image that have a greater impact on the model output. The heat maps before and after the model improvement are shown in Figure 4. Compared to Figure 4b, in Figure 4c, the color of the chili pepper targets is brighter. The improved module enhances the perception of the correct targets, better handling occluded targets, allowing for the model to more accurately focus on the features of the chili pepper fruit targets.

**Figure 5:**
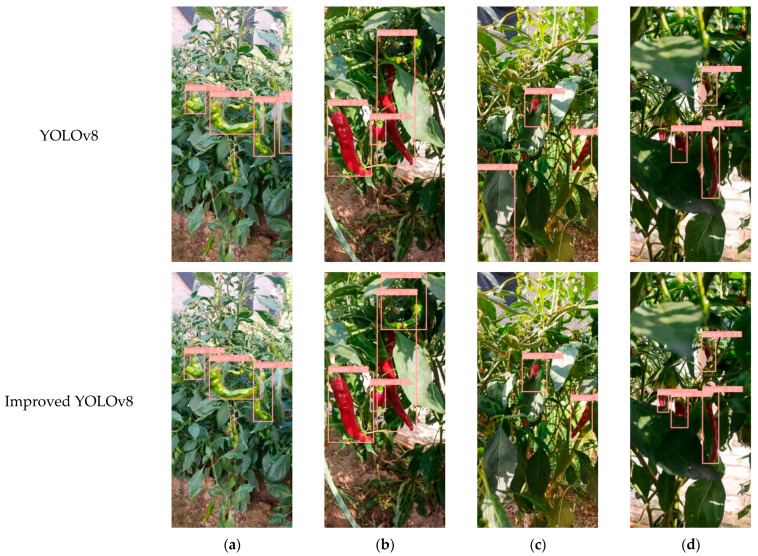
Comparison of detection effects of improved YOLOv8 and YOLOv8n. (**a**) Detection results of unobstructed chili pepper fruits; (**b**) Detection results of overlapping chili pepper fruits; (**c**) Detection results of chili pepper fruits obscured by leaves; (**d**) Detection results of mixed interference.

Improved YOLOv8 and YOLOv8n demonstrate different chili pepper detection results in various scenarios, as shown in Figure 5. In Figure 5a,c, it can be observed that the YOLOv8n model incorrectly identifies leaves as chili peppers, while the improved YOLOv8 model effectively avoids such false detections. In Figure 5b, the YOLOv8n model misses the chili pepper target in the upper right corner, and in Figure 5d, it also misses a distant chili pepper target. In contrast, the improved YOLOv8 model does not exhibit such missed detections in Figure 5b,d. These results indicate that the improved YOLOv8 model in this study can better focus on the features of chili pepper fruit targets, reducing both missed detections and false alarms.

## 3. Materials and Methods

### 3.1. Image Acquisition

**Figure 6:**
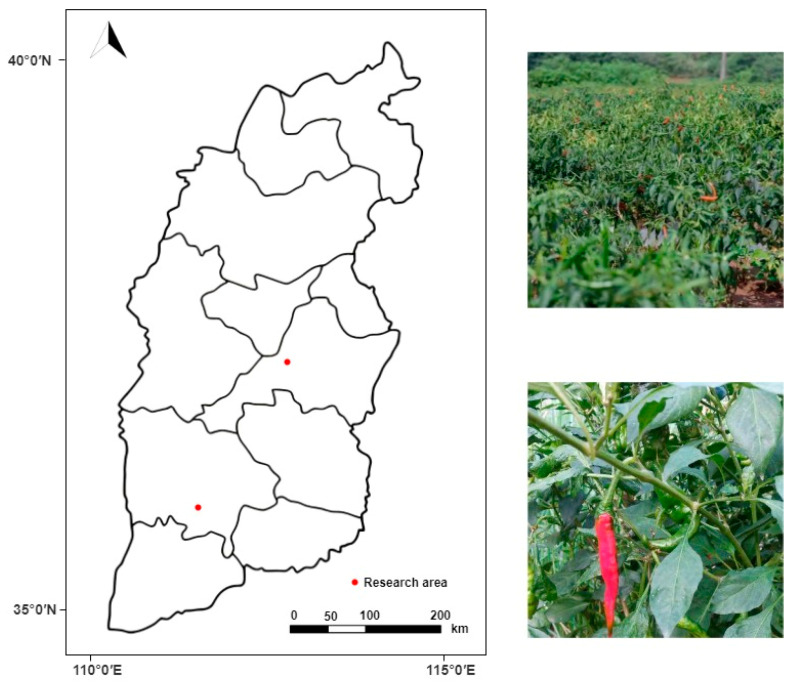
Sampling area map.

**Figure 7:**
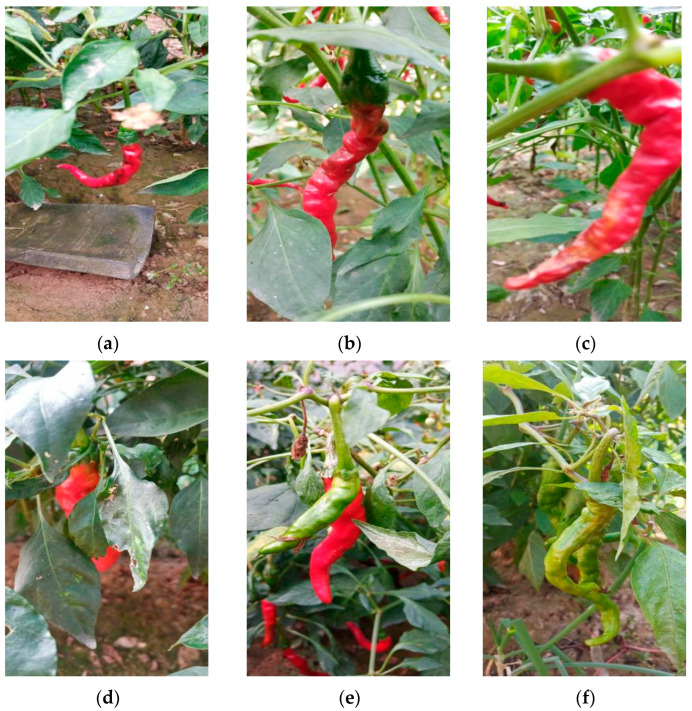
Different scenarios of chili pepper images: (**a**) unobstructed chili pepper fruits; (**b**) chili pepper fruits slightly obscured by leaves; (**c**) the chili pepper fruits are slightly obscured by branches; (**d**) the chili pepper fruits are heavily obscured by leaves; (**e**) the chili pepper fruits overlap; (**f**) mixed interference.

Pepper images for the experiment were collected from 1 to 7 October 2023 (8:00 to 18:00) in Xiangfen County, Linfen City, Shanxi Province, and Taigu District, Jinzhong City. The sampling locations are shown in Figure 6. Due to the complexity of natural environmental backgrounds and the diversity in pepper targets, different lighting conditions, shooting angles, and distances were selected during the collection process to further enhance the diversity in target data and the model’s generalization ability. Figure 7 shows some of the collected pepper images. The collected images have a resolution of 2160 × 4560, totaling 800 pepper images, including various scenes such as unobstructed fruits, fruits obstructed by leaves, fruits obstructed by branches, overlapping fruits, and mixed interference.

### 3.2. Data Filtering and Image Annotation

To improve the accuracy of pepper fruit recognition in natural environments, during the screening process, we first removed images of peppers that were partially damaged or blurred due to incomplete focusing of the shooting equipment, which are unavoidable artificial disruptions that interfere with the detection algorithm’s recognition. Additionally, to enhance the overall model’s robustness, when selecting pepper images, we specifically chose some images depicting natural situations where peppers were partially obscured by branches or leaves, as well as instances where peppers were overlapping each other. The retained images were clear pepper images captured from different angles, lighting conditions, and distances, making it easier for the YOLO object detection algorithm to learn the detailed features of the annotated pepper fruit targets and improve the overall model’s recognition accuracy. Through data filtering, a total of 427 high-quality images were obtained.

**Figure 8:**
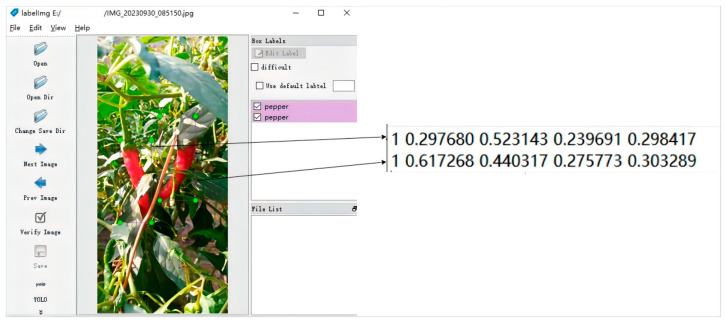
Labeling process.

Using LabelImg to annotate the obtained 427 images of pepper fruits, the pepper fruits were marked as “pepper”. We obtained a total of 875 annotations labeled “pepper”. The annotation process is shown in Figure 8, storing the chili pepper images with marked position and category information in the corresponding .txt files.

### 3.3. Dataset Augmentation

**Figure 9:**
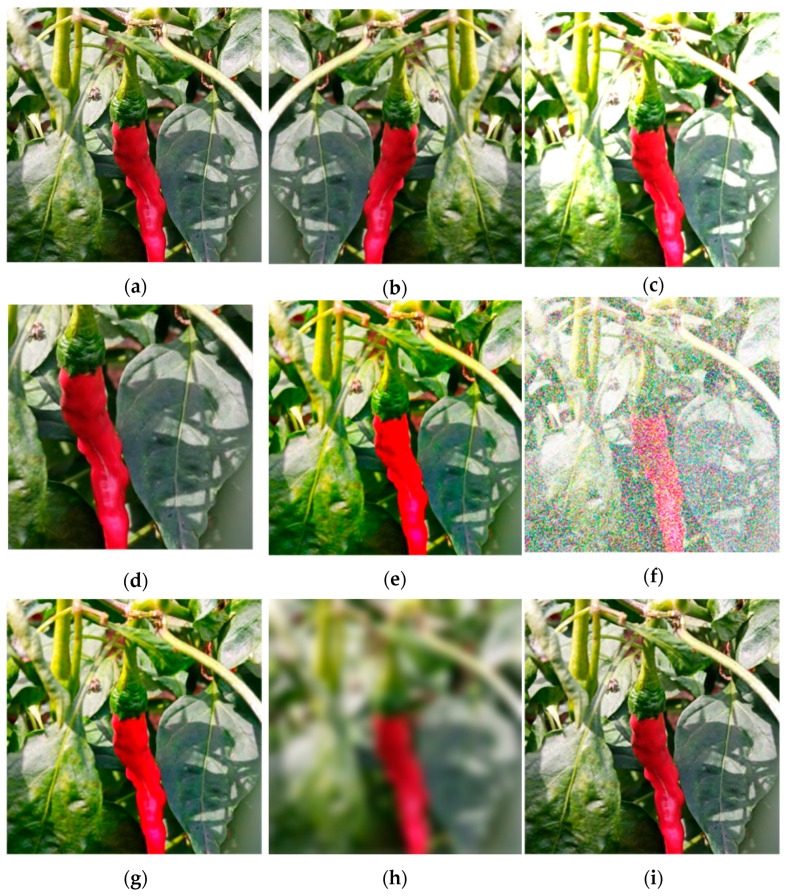
Chili pepper of data augmentation; (**a**) original image; (**b**) flipped image; (**c**) brightened image; (**d**) cropped image; (**e**) enhanced color image; (**f**) noisy image; (**g**) saturated image; (**h**) blurred image; (**i**) sharpened image.

To improve the robustness and generalization ability of the model, enriching the image data is necessary to better extract features of peppers in different scenes. This study employed data augmentation techniques, including horizontal flipping; adjusting brightness, color, and saturation; cropping; adding noise and Gaussian blur; and sharpening images. A total of 2135 pepper images were obtained using these methods. Examples of augmented images are shown in Figure 9.

The original dataset and the augmented dataset were split into training set, validation set, and test set in a ratio of 7:2:1. The data splitting results are shown in Table 6. The merged training set, validation set, and test set yielded a final training set of 1792 chili pepper images with 3666 “pepper” labels, a validation set of 513 chili pepper images with 1058 “pepper” labels, and a test set of 257 chili pepper images with 526 “pepper” labels.

### 3.4. Chili Pepper Object Detection Method

#### 3.4.1. YOLOv8 Network Structure

YOLOv8 is a SOTA (state-of-the-art) model developed by Ultralytics in January 2023. It inherits the advantages of the YOLO series while incorporating new features and improvements. Compared to its predecessor, YOLOv5, YOLOv8 has several key changes: (1) it replaces the C3 module in the backbone network with the C2f module; (2) it removes convolution operations during the upsampling process; (3) it adopts a decoupled head structure, separating the classification and detection tasks to further reduce model complexity.

**Figure 10:**
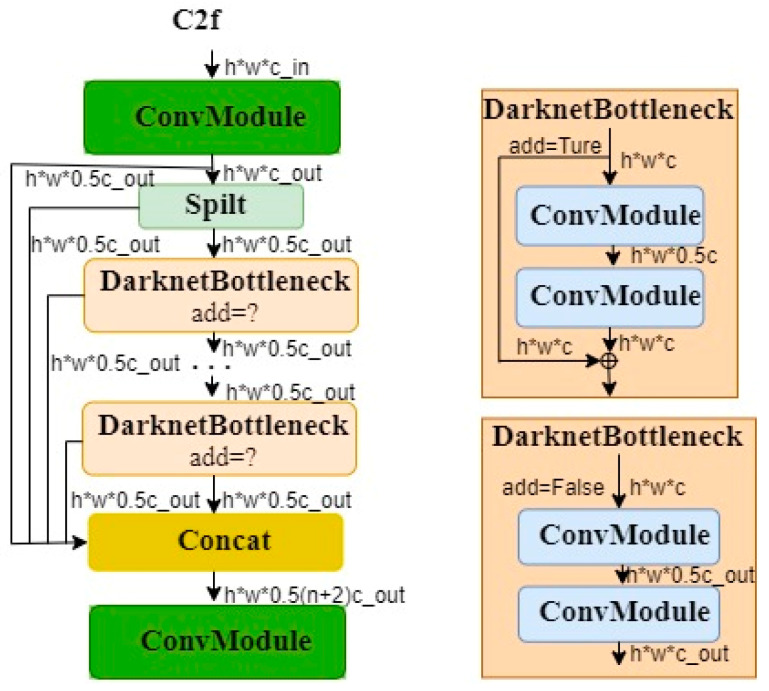
C2f structure.

The YOLOv8 network structure consists of four parts: input, backbone, neck, and head. The input part receives chili pepper images augmented with Mosaic data augmentation and feeds them into the network. The backbone uses the DarkNet53 structure to extract features from the bottom up and replaces the C3 module with the C2f module. The structure of the C2f module includes n bottleneck blocks, 2 convolutional layers, a split operation, and multiple skip connections, as shown in Figure 10.

**Figure 11:**
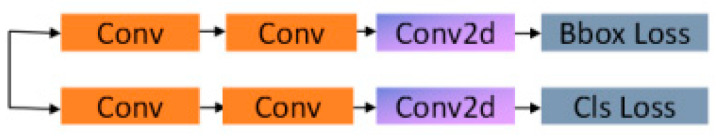
YOLOv8 head structure.

The head network mainly achieves feature fusion and target detection of peppers through the path aggregation network (PANet) and the detection head. PANet consists of feature pyramid networks (FPNs) and a PAN (bottom-up path aggregation). The FPN extracts feature layer information from top to bottom, fusing upper- and lower-level features to enhance the network’s ability to detect targets of different scales. The PAN extracts feature layer information from bottom to top to obtain positional information. The detection head outputs classification results and target coordinates through 3 branches of different sizes. The structure of a single detection head is shown in the figure, where the regression branch and prediction branch are separated. Information is first extracted separately through two 3 × 3 convolutions and one 1 × 1 convolution, and then Bbox loss and CLs loss are calculated, respectively, as shown in Figure 11.

#### 3.4.2. Improved YOLOv8 Network

**Figure 12:**
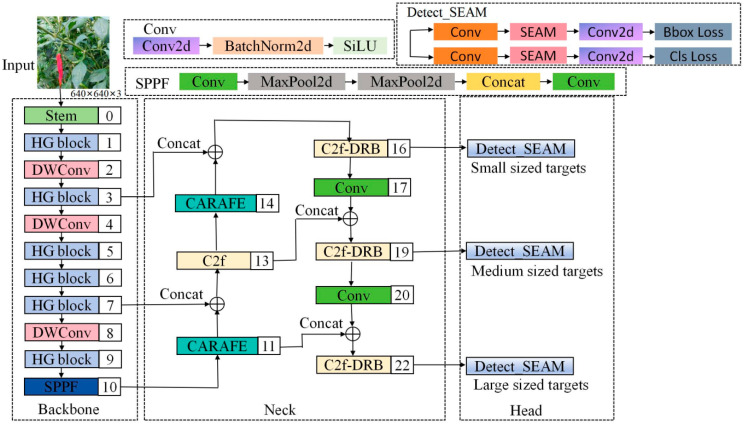
Improved YOLOv8 network structure. We replaced the improved HGNetV2 with the YOLOv8 backbone network and introduced the SEAM attention mechanism into the detection head. Some C2f layers were optimized to C2f-DRB. CARAFE upsampling operators replaced traditional upsampling operators. ConvModule stands for convolution + normalization + activation function; SPPF is the spatial pyramid pooling module; Maxpool2d is max pooling; Conv is convolution; Contact is the feature concatenation module; Bbox Loss and Cls Loss refer to bounding box loss and classification loss. respectively.

In natural environments, chili pepper fruits are prone to occlusion, requiring models to extract image feature information from different scales and levels to identify objects of varying sizes and shapes. The traditional YOLOv8n model uses simplistic feature extraction and fusion methods in its backbone and head, making it challenging to fully utilize feature information across different scales. Therefore, to ensure that the recognition algorithm for automated chili pepper picking maintains high accuracy and real-time performance in complex, unstructured environments, this study optimizes and improves the YOLOv8n model in the following four aspects: First, to accelerate network detection speed, the backbone network is redesigned, replacing it with a more lightweight and improved HGNetV2. Second, to enhance the network’s ability to recognize occluded images, an effective SEAM attention mechanism for occluded images is introduced into the network’s detection head. Third, the redundancy in the C2f network structure is analyzed, and to reduce the model’s floating-point operations and computational load, some C2f structures are optimized. Lastly, CARAFE upsampling operators are introduced to replace traditional upsampling methods, further improving detection accuracy. This paper presents an improved YOLOv8 structure, as shown in Figure 12, replacing the enhanced HGNetV2 as the backbone network, introducing the SEAM attention mechanism into the detection head, optimizing parts of the C2f structure, and using the CARAFE upsampling operator instead of traditional upsampling to effectively enhance model detection capability and speed while reducing missed detections and false alarms. When chili pepper images are inputted into this model, they undergo feature extraction through the HGNetV2 network first, aiming to achieve high detection accuracy and speed with minimal model parameters. Subsequently, feature fusion is conducted via the neck network, followed by the detection head integrated with the SEAM attention mechanism to further enhance the model’s ability to handle occluded chili pepper features, thereby improving the overall network’s expressive power.

This article focuses on improvements in four main aspects:

(1) Improved HGNetV2 Model

RT-DETR is the first real-time vision transformer (ViT) model introduced by Baidu, claiming superiority over the YOLO series in real-time detection. It utilizes two backbones, one being HGNet and the other ResNet. The HGNetV2 network is more lightweight and accurate. Therefore, this study attempts to replace the YOLOv8 backbone with HGNetV2 to meet the real-time and efficient requirements for pepper fruit picking.

**Figure 13:**
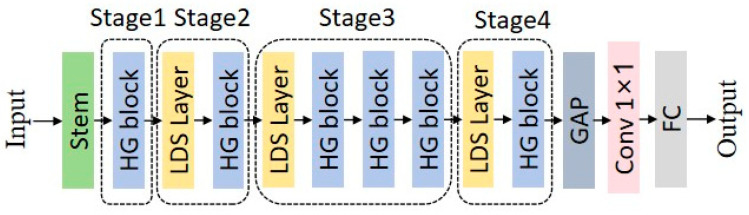
HGNetV2 structure.

The main idea of the HGNetV2 model is to extract features in a hierarchical manner, where complex patterns can be learned at different scales and abstraction levels, enhancing the network’s ability to process complex image data. This hierarchical and efficient processing is particularly beneficial for tasks like image classification. The overall structure of the HGNetV2 model backbone network is shown in Figure 13:

1. Stem layer: This is the initial preprocessing layer of the network, typically consisting of convolutional layers to start extracting features from the raw input data.
2. HG block (hierarchical graph block): These blocks are the core components of the network, designed to process data hierarchically. Each HG block may handle data at different abstraction levels, allowing for the network to learn from low-level and high-level features.
3. LDS (learnable downsampling) layer: These layers located between HG blocks may perform downsampling operations, reducing the spatial dimensions of the feature maps, decreasing computational load, and potentially increasing the receptive field of subsequent layers.
4. GAP (global average pooling): Before final classification, the GAP layer reduces the spatial dimensions of the feature maps to a single vector per feature map, aiding in improving the network’s robustness to spatial transformations of input data.
5. Final convolution and fully connected (FC) layers: These include a 1 × 1 convolutional layer to combine features and fully connected layers to map these features to the required number of output classes.

**Figure 14:**
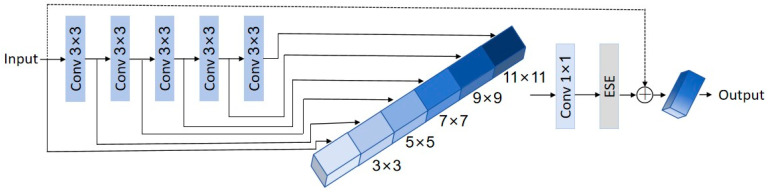
HG block structure.

The HGNetV2 is composed of multiple HG blocks, the structure of which is shown in Figure 14. It includes several convolutional layers with different filter sizes to capture diverse features.

From the above HGNetV2 model structure, it can be seen that there is a large number of convolution operations in this network. Therefore, we can optimize HGNetV2 by using a more lightweight depthwise separable convolution, DWConv (depthwise convolution).

DWConv, a lightweight convolution operation proposed by Google in 2018, achieves comparable effects to traditional convolutions with fewer parameters and computational complexity. It is widely used in deep learning models on mobile and edge devices. The main difference between DWConv and traditional convolutions lies in the separation of its convolution kernel, which is divided into two parts corresponding to the depth and spatial dimensions. For each input channel, DWConv performs convolution with a kernel equal to the number of channels, and then aggregates the results along the depth dimension to obtain the final output. This operation can maintain output quality with a small number of convolution kernels, thus reducing parameters and computational complexity. Specifically, for a convolution kernel of size k, the parameter count is k ∗ k ∗ c_in ∗ c_out, where c_in represents the number of input channels and c_out represents the number of output channels. With DWConv, the parameter count is reduced to k ∗ k ∗ c_in since different channels share the same convolution kernel. This reduction in parameter count can improve the efficiency of deep learning models on mobile and edge devices.

**Figure 15:**
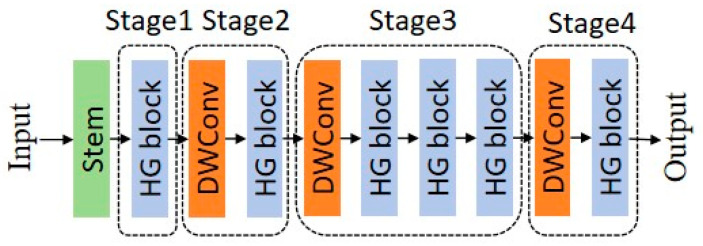
Improved HGNetV2 structure.

The improved HGNetV2 model is presented in Figure 15.

(2) SEAM Attention Mechanism

**Figure 16:**
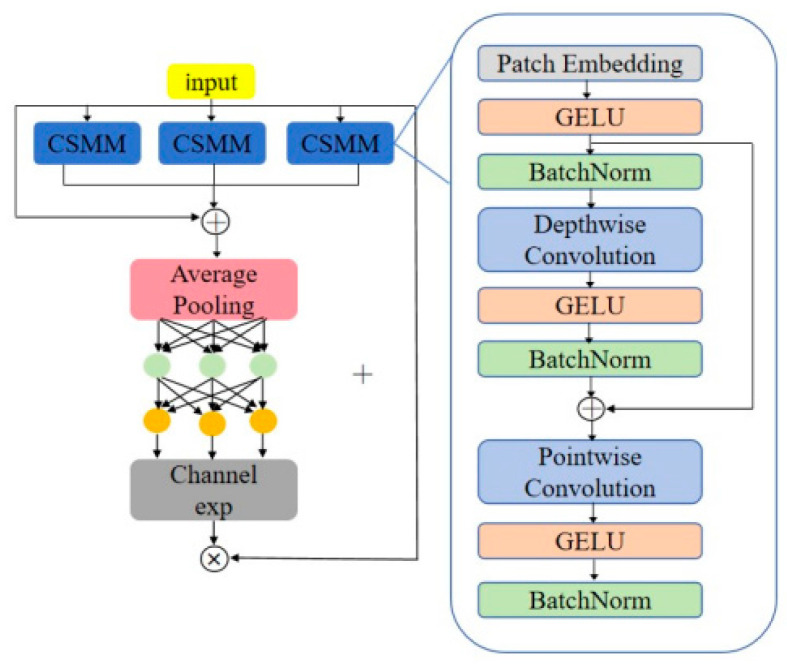
SEAM structure.

The SEAM (spatially enhanced attention module) is an attention mechanism proposed by YOLO-Face to improve object occlusion detection. Its goal is to compensate for the loss of response from occluded objects by enhancing the response of unoccluded objects. The SEAM aims to improve object occlusion detection by learning the relationship between occluded and unoccluded areas. The structure of the SEAM is as shown in Figure 16: The first part of SEAM consists of depthwise separable convolution with residual connections. Depthwise separable convolution operates layer by layer, separating the convolution channel by channel. While depthwise separable convolution can learn the importance of different channels and reduce the number of parameters, it ignores the information relationship between channels. To compensate for this loss, the outputs of different depth convolutions are subsequently combined through pointwise (1 × 1) convolution. Then, a two-layer fully connected network is used to merge information from each channel, enabling the network to enhance connections between all channels. It is hoped that this model can compensate for the loss mentioned above in occlusion scenarios by learning the relationship between occluded and unoccluded pepper fruits in previous steps. Next, the output learned by the fully connected layers is processed by an exponential function to expand the value range from [0,1] to [1,e]. This exponential normalization provides a monotonic mapping relationship, making the result more tolerant of position errors. Finally, the output of the SEAM is used as attention multiplied by the original feature, allowing for the model to more effectively handle occlusions of pepper fruits and diminish background areas.

**Figure 17:**
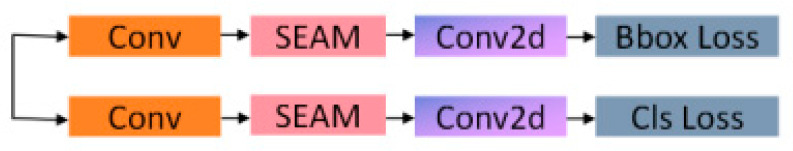
Improved YOLOv8 head.

During the pepper harvesting process, it is common for fruits to be occluded. In order to improve the capability of extracting pepper target features under such circumstances, this study integrates the SEAM attention mechanism into the YOLOv8 detection head. The 3 × 3 convolutional layers in the YOLOv8 detection head are replaced with SEAMs, as illustrated in Figure 17. With this improvement, the YOLOv8 detection head is capable of multi-scale pepper detection, placing greater emphasis on the pepper fruit regions in the image while weakening background areas. Additionally, the original 3 × 3 convolutional layers in the YOLOv8 detection head would increase the computational load of the module. By substituting the 3 × 3 convolutional layers with SEAMs, it is possible to further reduce the floating-point operations and computational load during the convolution process.

(3) Dilated Reparam Block

**Figure 18:**
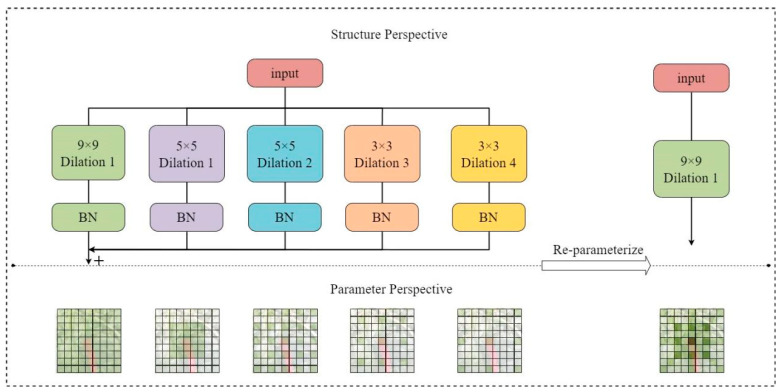
Dilated reparam block network architecture.

The UniRepLKNet model innovatively reconsiders the design of using multiple small convolutional kernels in traditional models by introducing an architecture based on large convolutional kernels. One significant innovation is the introduction of the dilated reparam block module, which reparameterizes convolutional layers with large kernels to enhance performance without increasing inference costs. Reparam block uses dilated small-kernel convolution layers to enhance undilated large-kernel layers. From a parameter perspective, these dilated layers are equivalent to undilated convolution layers with larger sparse kernels; thus, the entire block can effectively be seen as transforming into a large-kernel convolution layer. The structure of dilated reparam block is shown in Figure 18.

**Figure 19:**
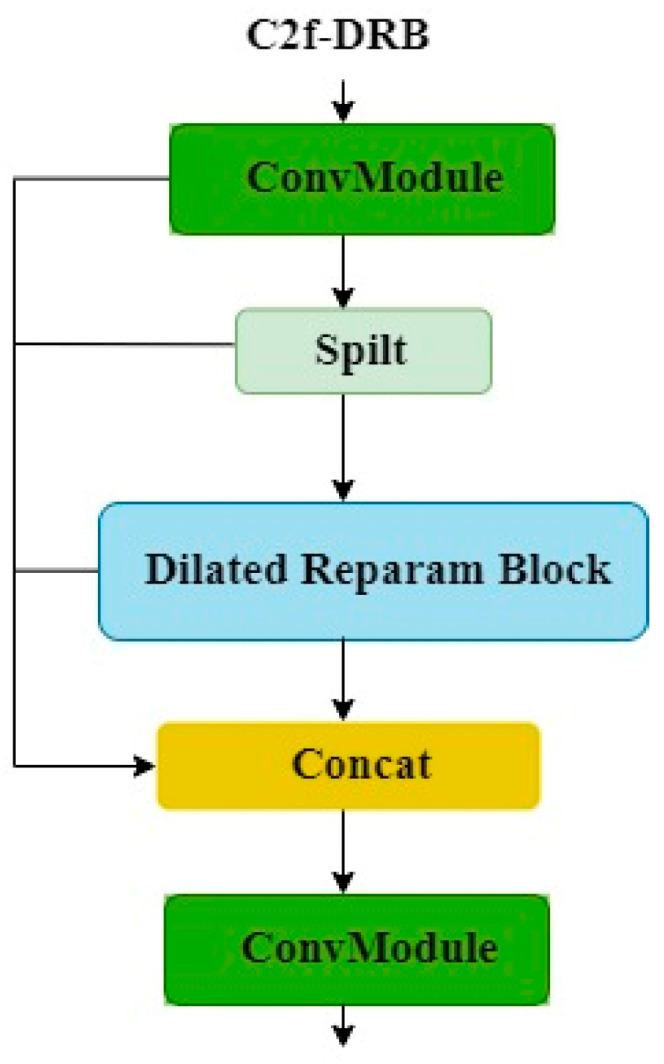
C2f-DRB network architecture.

YOLOv8’s C2f module employs numerous bottleneck structures, which extract more features but lead to excessive redundancy in channel information, resulting in increased computational overhead. This paper utilizes the dilated reparam block from UniRepLKNet, integrating it into the feature extraction C2f module through a class inheritance approach, replacing the bottleneck as the primary gradient flow branch. This reduces model parameters and computational costs. The new module is named C2f-DRB. The structure of the C2f-DRB module is shown in Figure 19.

(4) CARAFE

The CARAFE (content-aware reassembly of features) upsampling operation has a large receptive field during reassembly, guiding the reassembly process based on input features, while keeping the operator itself lightweight.

CARAFE consists of two steps: In the first step, a reassembly kernel is predicted for each target position based on the content of the input. The second step involves reassembling features using the predicted kernels. Assuming a feature map X of size C × H × W and an upsampling rate σ (assumed to be an integer), CARAFE generates a new feature map X’ of size C × σH × σW. For any target position l′ = (i′,j′) in the output X’, there exists a corresponding source position l = (i, j) in the input X, where i = i′/σ, j = j′/σ.

In the first step, the kernel prediction module Ψ predicts an appropriate kernel W_l’_ for each position l’ based on the neighborhood of X_l_, as shown in Equation (1). The reassembly step in the second step is formulated as shown in Equation (2), where Ψ is the content-aware reassembly module that reassembles the neighborhood of X_l_ with kernel W_l’_.

(1) $$W_{l^{\prime }}=\psi (N(X_{l},\;\mathrm{kencoder}))$$

(2) $$X_{l^{\prime }}^{\prime }=\phi (N(X_{l},\;\mathrm{kup}),W_{l^{\prime }})\;$$

Here, N(X_l_, k) represents the k × k sub-region of X centered at position l, denoted as X_l’_s neighborhood.

### 3.5. Training Environment and Evaluation Indicators

The operating system used for the experiment was Windows 10. The CPU model was an Intel(R) Core(TM) i7-13700F @2.10GHz. The GPU model was a NVIDIA GeForce RTX 4080. The system had 32GB of RAM and a 1TB mechanical hard drive. The programming language used was Python 3.9. The deep learning framework used was PyTorch 2.0.1. The GPU acceleration libraries used were CUDA 11.8 and CUDNN 8.8.0. Training parameters are shown in Table 7.

The detection process of chili peppers requires consideration of both detection accuracy and speed. For model detection accuracy, precision (P), recall (R), and mean average precision (mAP) were chosen as evaluation metrics. For model detection performance, model parameters, model size, GFLOPs, and FPS were selected as evaluation metrics.

The detection process for chili fruits needs to consider both detection accuracy and speed. Regarding model detection accuracy, evaluation metrics such as precision (P), recall (R), F-score, and mean average precision (mAP) are utilized, with calculation formulas given as in Equations (3)–(6).

(3) $$P=\frac{\mathrm{TP}}{\mathrm{TP}+\mathrm{FP}}$$

(4) $$R=\frac{\mathrm{TP}}{\mathrm{TP}+\mathrm{FN}}$$

(5) $$\mathrm{mAP}=\frac{\sum_{i=1}^{C}{\mathrm{AP}}_{i}}{C}$$

where TP represents the number of true positive samples, FP denotes the number of negative samples incorrectly labeled as positive, and FN indicates the number of positive samples missed during detection. C represents the total number of classes, where in this experiment, for chili pepper object detection, C = 1. ${\mathrm{AP}}_{i}$ denotes the area under the precision–recall curve for class i, specifically indicating the area under the precision–recall curve computed for chili pepper object detection in this experiment.

The F-score ($F_{\beta -\mathrm{score}}$) is a comprehensive metric that evaluates both P and R, calculated as shown in Equation (6). In practical applications of harvesting robotic arms, to prevent erroneous identification from damaging fruit trees, precision in target recognition is considered more important than recall. Therefore, in this paper, the weighting factor β is set to 0.5. In contrast to the commonly used F1 score, the importance of P is twice that of R, emphasizing precision.

(6) $$F_{\beta \text{-}\mathrm{score}}=(1+\beta^{2})\frac{P\times R}{\beta^{2}\times P+R}$$

In terms of model lightweighting, parameter count, model memory footprint, and GFLOPs are chosen as evaluation metrics.

## 4. Discussion

**Figure 20:**
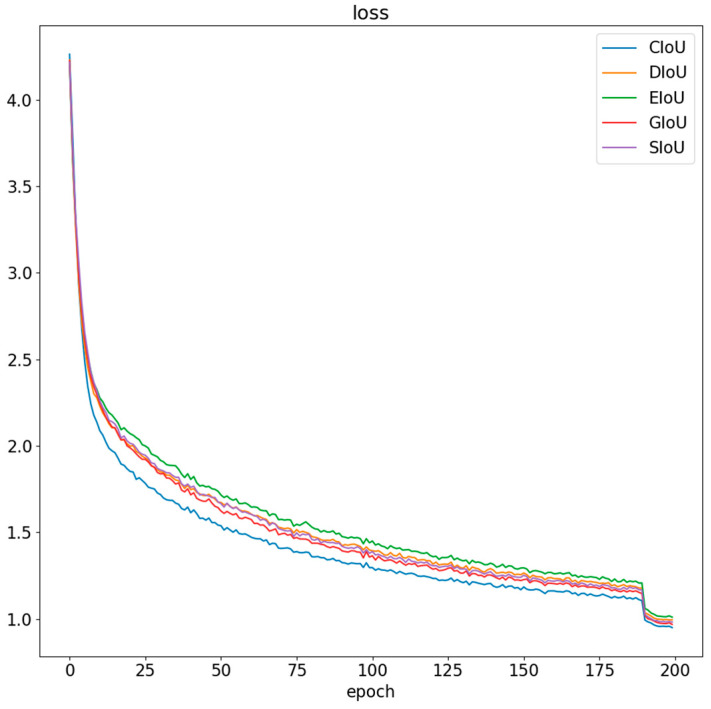
Comparison of loss function.

(1) The YOLOv8 object detection model uses CIoU as the bounding box regression loss function. In order to further explore the impact of loss functions on the improved YOLOv8 model in this paper, this experiment compared the convergence of the improved YOLOv8 model when using DIoU, GIoU, EIoU, SIoU, and CIoU loss functions. The experimental results are shown in Figure 20. As shown in Figure 20, the convergence speed is slowest when using EIoU, while DIoU, SIoU, and GIoU converge slightly faster than EIoU and have lower loss values after convergence. The CIoU loss function has the fastest convergence speed, and the loss value after convergence is lower than the other four loss functions. Therefore, the improved YOLOv8 model in this paper still uses CIoU as the bounding box regression loss function.

However, in dense object detection tasks, the predicted boxes may overlap with each other, leading to errors in the calculation of the overlapping area using CIoU, which can affect detection accuracy. Since this dataset includes relatively few dense scenes of chili peppers, CIoU performs well. In the future, for dense object data scenarios, further optimization of the loss function may be considered.

(2) To improve the detection accuracy of YOLO series models, many researchers have introduced the attention mechanism into YOLO models and achieved good results. Chen Yu [36] et al. integrated the CA (coordinate attention) mechanism into the feature extraction network of YOLOv5s, achieving the extraction of features related to tea diseases. Li et al. [37] improved the backbone network of YOLOv8 using the MHSA attention mechanism to enhance the network’s ability to extract diverse features, establishing a model for grading and counting tomato ripeness. In natural environments, chili fruits are easily obstructed by branches and leaves, making it difficult for harvesting robots to pick them. The SEAM attention mechanism can better focus on obstructed targets. Therefore, this study attempts to integrate the SEAM attention mechanism into the YOLOv8 detection head to enhance the network’s feature extraction capability. Experimental results confirm the effectiveness of this approach.

(3) We attempted to enhance traditional upsampling methods using CARAFE and DySample sampling techniques. The experimental results are shown in Table 8. Analysis of the experimental results from the table reveals that while CARAFE shows increases in size, GFLOPs, and parameter count, it achieves improvements in F_0.5-score_, mAP_0.5_, and mAP_0.5:0.95_ by 0.13, 0.1, and 1.4 percentage points, respectively. However, the DySample upsampling operator performs poorly on this dataset, leading to varying degrees of negative impact on F_0.5-score_, mAP_0.5_, and mAP_0.5:0.95_. Therefore, this study ultimately adopts the CARAFE upsampling operator to enhance pepper detection accuracy.

## 5. Conclusions

In order to address challenges in pepper fruit detection under natural environments, such as interference from complex backgrounds and fruit occlusions, this study proposes a lightweight pepper detection model. Based on YOLOv8n, the backbone network was replaced with the HGNetV2 model to hierarchically extract features, ensuring lightweight design while maintaining detection accuracy. The convolutional layers in the Head network were substituted with SEAM attention mechanisms to enhance detection capabilities for occluded pepper fruits. The dilated reparam block was optimized for parts of the C2f structure, and the CARAFE upsampling operator replaced traditional upsampling, further improving model detection performance. Experimental results show that the refined model achieved increases of 2.5, 0.1, 1.98, 2, and 5.2 percentage points in P, R, F_0.5-score_, mAP_0.5_, and mAP_0.5:0.95_, respectively, achieving 97.8%, 91.5%, 96.47%, 96.3%, and 79.4%. The model size is 4.6 MB. Compared to the baseline YOLOv8n network, the improved model reduced parameters and GFLOPs by 29.5% and 28.4%, respectively. Compared to mainstream lightweight object detection networks like YOLOv5n, YOLOv6n, YOLOv7-tiny, YOLOv9, and YOLOv10, the proposed model also demonstrates significant advantages.

However, the improved YOLOv8 model presented in this paper has not yet been applied on embedded platforms. In future practical deployments, we will evaluate the model’s effectiveness and practicality in natural environments for harvesting, continue to optimize the detection model, and enhance detection efficiency.

## Figures and Tables

**Table 1:** Comparison of different models.

Model	P/%	R/%	F_0.5-score_/%	mAP_0.5_/%	mAP_0.5:0.95_/%	Model Size/MB	GFLOPs	Parameters /10^6^
YOLOv5n	93.6	85.8	91.93	94	70.1	5.3	7.1	2.5
YOLOv6n	95.9	88.3	94.28	94.9	74.1	8.7	11.8	4.2
YOLOv7-tiny	90.5	82.2	88.71	89.6	57.7	12.2	13.2	6.0
YOLOv8n	95.3	91.4	94.49	94.3	74.2	6.3	8.1	3.0
YOLOv9	94.9	89.8	93.83	95.3	72.1	51.6	102.3	25.3
YOLOv10	92.6	80.3	89.85	91	69	5.7	8.2	2.7
Faster R-CNN	50.61	93.3	55.71	90.1	43.2	108	370.21	137
RT-DETR	94.2	93.1	93.98	96.1	74.6	59.1	100.6	28.4

**Table 2:** Improved YOLOv8 and YOLOv8n detection results comparison.

Model	P/%	R/%	F_0.5-score_/%	mAP_0.5_/%	mAP_0.5:0.95_/%	Model Size/MB	GFLOPs	Parameters/10^6^
Improved YOLOv8	97.8	91.5	96.47	96.3	79.4	4.6	5.8	2.1
YOLOv8	95.3	91.4	94.49	94.3	74.2	6.3	8.1	3.0

**Table 3:** Results of ablation test.

Experiment Number	Baseline	Improved HGNetV2	SEAM	DRB	CARAFE	F_0.5-score_/%	mAP/%	mAP_0.5:0.95_/%	Parameters	Model Size/MB	GFLOPs
1	√	×	×	×	×	94.49	94.3	74.2	3,006,038	6.3	8.1
2	√	√	×	×	×	93.64	96.2	77.9	2,351,290	5	6.9
3	√	×	√	×	×	93.0	94.8	74.3	2,817,878	5.9	7.0
4	√	×	×	√	×	95.24	95.4	75.5	2,823,478	5.9	7.8
5	√	×	×	×	√	95.02	95.4	75.4	3,146,142	6.5	8.4
6	√	√	√	×	×	95.46	96.7	79.6	2,163,130	4.7	5.8
7	√	√	√	√	×	96.34	96.3	78.3	1,980,570	4.3	5.5
8	√	√	√	√	√	96.47	96.3	79.4	2,120,674	4.6	5.8

**Table 4:** Comparison of different backbone networks.

Backbone Network	P/%	R/%	mAP/%	Model Size/MB	GFLOPs	Parameters
EfficientViT	96.5	91.9	96.3	8.8	9.4	4,008,662
Fasternet	98.5	92.8	97.1	8.6	10.7	4,172,570
EfficientFormerV2	98.8	92.4	97.3	41.4	11.7	5,105,790
HGNetV2	97.6	91.1	96.5	5.0	6.9	2,351,290

**Table 5:** C2f optimization results.

Trial Number	Position in YOLOv8n	Replaced Module	P/%	R/%	F_0.5-score_/%	GFLOPs	Parameters
1			96.2	92.6	95.46	5.8	2,163,130
2	16	C2f-DBB	94.9	91.1	94.11	5.8	2,163,130
3	16	C2f-ODConv	97	91.1	95.76	5.6	2,173,214
4	16	C2f-FasterBlock	95.6	90.1	94.45	5.7	2,149,370
5	16	C2f-DRB	97.2	91.5	96.00	5.7	2,155,482
6	16/19	C2f-DRB	96.7	90.5	95.39	5.6	2,121,754
7	16/19/22	C2f-DRB	97.6	91.6	96.34	5.5	1,980,570

**Table 6:** Distribution of chili pepper.

Data	Category	Training Set	Validation Set	Test Set	Total
Original dataset	Chili pepper image	298	86	43	427
	“pepper” label	616	174	85	875
Augmented dataset	Chili pepper image	1494	427	214	2135
	“pepper” label	3050	884	441	4375

**Table 7:** Training parameters.

Training Parameters	Values
Initial learning rate	0.01
Optimizer	SGD
Optimizer momentum	0.937
Optimizer weight decay rate	0.0005
Number of image per batch	16
Number of epochs	200

**Table 8:** Comparison of different sampling methods.

Model.	F_0.5-score_/%	mAP_0.5_/%	mAP_0.5:0.95_/%	Model Size/MB	GFLOPs	Parameters
Upsample	96.34	96.2	78.3	4.3	5.5	1,980,570
CARAFE	96.47	96.3	79.4	4.6	5.8	2,120,674
DySample	96.27	95.8	78.2	4.3	5.5	1,992,922

## Data Availability

The research project is ongoing, and part of the data is available upon request. The code can be requested from the first author.
